# Greater dapivirine release from the dapivirine vaginal ring is correlated with lower risk of HIV‐1 acquisition: a secondary analysis from a randomized, placebo‐controlled trial

**DOI:** 10.1002/jia2.25634

**Published:** 2020-11-18

**Authors:** Elizabeth R Brown, Craig W Hendrix, Ariane van der Straten, Flavia M Kiweewa, Nyaradzo M Mgodi, Thesla Palanee‐Philips, Mark A Marzinke, Linda‐Gail Bekker, Lydia Soto‐Torres, Sharon L Hillier, Jared M Baeten, the MTN‐020/ASPIRE Study Team, Thesla Palanee‐Phillips, Katie Schwartz, Francis Martinson, Vaneshree Govender, Samantha Siva, Zakir Gaffoor, Logashvari Naidoo, Arendevi Pather, Nitesha Jeenarain

**Affiliations:** ^1^ Vaccine and Infectious Disease and Public Health Sciences Divisions Fred Hutchinson Cancer Research Center Seattle WA USA; ^2^ Department of Biostatistics University of Washington Seattle WA USA; ^3^ Department of Medicine Johns Hopkins University Baltimore MD USA; ^4^ Women's Global Health Imperative RTI International San Francisco CA USA; ^5^ Makerere University‐Johns Hopkins University Research Collaboration Kampala Uganda; ^6^ University of Zimbabwe College of Health Sciences Clinical Trials Research Centre Harare Zimbabwe; ^7^ Wits Reproductive Health and HIV Research Institute School of Clinical Medicine University of the Witswatersrand Johannesburg South Africa; ^8^ Desmond Tutu HIV Foundation Clinical Research Site Cape Town South Africa; ^9^ Division of AIDS National Institute of Allergy and Infectious Diseases National Institutes of Health Bethesda MD USA; ^10^ Department of Obstetrics, Gynecology and Reproductive Sciences University of Pittsburgh School of Medicine and Magee‐Womens Research Institute Pittsburgh PA USA; ^11^ Departments of Medicine, Epidemiology, and Global Health University of Washington Seattle WA USA

**Keywords:** adherence, clinical trials, HIV prevention, women, HIV prevention trials, Africa

## Abstract

**Introduction:**

A vaginal ring containing 25 mg of the antiretroviral dapivirine has demonstrated efficacy in reducing women’s risk of sexually acquiring HIV‐1; however, imperfect ring use likely diluted efficacy estimates in clinical trials. The amount of dapivirine remaining in returned rings may reflect the extent of product use, permitting estimation of HIV protection in the context of consistent use.

**Methods:**

We measured the amount of dapivirine in returned rings from a placebo‐controlled trial of the dapivirine vaginal ring conducted between August 2012 and June 2015 among 2629 African women. Phase I/II studies established that greater than 4 mg of dapivirine on average is released from the ring when used consistently over 28 days and ≤0.9 mg released suggested non‐use. We assessed the relative risk reduction associated with levels of ring use using residual dapivirine in returned rings as a time‐dependent covariate for HIV‐1 infection in multivariable Cox models, including multiple exploratory analyses designed to estimate upper limits of efficacy given uncertainty in timing of HIV‐1 acquisition. All models were adjusted for baseline covariates associated with HIV risk and adherence.

**Results:**

Residual dapivirine levels indicating at least some use (>0.9 mg released over a month) were associated with a 48% relative reduction in HIV‐1 acquisition risk (95% confidence interval (CI): 21% to 66%; *p* = 0.002) compared to the placebo. Exploratory analyses accounting for potential misclassification in timing of HIV‐1 acquisition estimated 75% to 91% HIV‐1 risk reduction with> 4 mg released when compared to placebo. Results limited to the subgroup of women <25 years of age, who tended to have lower adherence, were generally consistent to those overall.

**Conclusions:**

Residual dapivirine levels, an objective measure of adherence, were correlated with HIV‐1 protection in a secondary analysis of a randomized trial. Periods of ring use were associated with approximately 50% protection, with exploratory analyses suggesting higher protection with more consistent use. The dapivirine vaginal ring is the first method to fulfil the promise of a fully reversible, long‐acting, woman‐initiated approach for discreet HIV‐1 prevention.

## 1 INTRODUCTION

Worldwide, nearly 870 000 women and girls are infected with HIV‐1 every year [1], with the vast majority of infections occurring in sub‐Saharan Africa. New, effective and acceptable HIV‐1 prevention tools, controlled by women, are urgently needed. The use of antiretroviral medications as pre‐exposure prophylaxis (PrEP), formulated first as tenofovir‐containing oral pills and more recently into longer acting sustained‐release approaches, has emerged as a powerful HIV‐1 prevention strategy for men and women worldwide [2, 3, 4]. A vaginal ring containing the antiretroviral non‐nucleoside reverse transcriptase inhibitor dapivirine was well tolerated and reduced HIV‐1 acquisition risk in two phase III trials and their open label extensions and is a promising new woman‐controlled, sustained‐release PrEP approach [5, 6, 7, 8].

In several clinical trials of oral pills as PrEP, initial analyses were based on intention‐to‐treat principles; however, due to objective evidence that an important fraction of subjects was not adherent to the study medication, additional analyses based on objective, biologic measures of use were done to estimate HIV‐1 protection among adherent individuals [9, 10, 11]. Those adherence‐based analyses found greater HIV‐1 protection when limited to subjects with objective evidence of product use, as defined by measurement of the antiretroviral in participant samples including plasma, dried blood spots and hair. In fact, estimates of HIV‐1 risk reduction derived from secondary analyses of high PrEP adherence are cited by normative guidelines and public health agencies as the level of HIV‐1 protection that potential PrEP users should expect [12].

The quantity of dapivirine remaining in returned vaginal rings provides an objective measure of adherence to that PrEP product. Average release levels over a month of use were assessed in phase I studies [13, 14] and product manufacturing and assay parameters define rings that have had little or no use. However, the relationship between residual dapivirine ring levels and HIV‐1 protection has not been assessed. In analyses analogous to those that interrogated the protective effectiveness of tenofovir‐based oral PrEP, we assessed the relationship between residual dapivirine levels and incident HIV‐1 in a placebo‐controlled trial of the dapivirine vaginal ring, with a goal of refining estimates of efficacy that might be conferred by consistent use of the ring, defined as continuous use of the ring over its prescribed period of 28 days.

## 2 METHODS

### 2.1 Population and procedures

MTN‐020/ASPIRE was a multi‐centre, randomized, double‐blind, placebo‐controlled phase III trial of the dapivirine vaginal ring [5]. The primary analysis was intention‐to‐treat, comparing the HIV‐1 incidence rate among those assigned to the active dapivirine vaginal ring arm to that among those assigned to the placebo arm, regardless of actual use of the study product. In that analysis, HIV‐1 protection effectiveness was 27% (95% confidence interval [CI]: 1, 46). Pre‐defined secondary analyses showed greater HIV‐1 protection in some subgroups, including women ≥ 25 years of age.

Between August 2012 and June 2015, 2629 healthy, sexually active, non‐pregnant, HIV‐1 seronegative women aged 18 to 45 years were enrolled and followed at 15 research sites in Malawi, South Africa, Uganda and Zimbabwe [5, 15]. At enrolment, women were randomized to receive a vaginal ring containing either 25 mg of dapivirine or a placebo ring containing no dapivirine; rings were to be worn continuously for 28 days at a time. Women were scheduled for visits every 28 days that included HIV‐1 antibody testing and exchange of a used ring for a new ring; visits were considered on schedule if they occurred between 21 and 35 days, although visits occurring after 35 days were completed for participant convenience. At quarterly visits, plasma was collected and archived; for women who seroconverted to HIV‐1, back testing of plasma samples for HIV‐1 RNA was performed to better define the timing of HIV‐1 acquisition. Therefore, the most precise timing of infection and dapivirine ring levels aligned at quarterly visits. Planned follow‐up was for a minimum of 12 months. The study protocol was approved by institutional review boards associated with each study site and was registered with ClinicalTrials.gov (NCT016170096). All participants provided written informed consent.

### 2.2 Measurement of residual dapivirine levels in returned rings

Beginning one year into the trial, returned rings were collected and tested for remaining dapivirine levels; prior to that time, returned rings were discarded. Testing for remaining dapivirine was performed using acetone extraction and high‐pressure liquid chromatography at a commercial laboratory (Parexel) located in Bloemfontein, South Africa. All returned rings were from the active arm and a subset from the placebo arm were sent to the lab for testing starting one year into the study. One or two unused rings with known load level were included In each assay batch for returned rings. The standard deviation in released DPV measures from these rings was used to estimate measurement error. Plasma, collected at quarterly visits, was also tested for dapivirine using previously described liquid chromatographic‐tandem mass spectrometric methods [16] in a US laboratory masked to the residual drug assay results; however, because plasma concentrations rise to near‐steady‐state by approximately eight hours post‐ring insertion and do not reflect long‐term ring use, plasma measurements were not used as the key measure of adherence for the present analysis.

Ideally, the level of dapivirine in the returned rings can identify consistent use of the ring, that is rings that are worn continuously for 28 days as intended. Interpretation of the quantity of remaining dapivirine in returned rings was guided by data from phase I studies, which established that on average >4 mg is released with consistent use of the ring [17]. To account for the fact that not all visits occurred exactly spaced by 28 days, dapivirine levels were analysed as a ratio of the amount released (manufacturing load level minus remaining dapivirine) to the number of days since its dispensation. Thus, this measure is analogous to an average rate of dapivirine release per day that the woman had the ring in her possession, and higher levels of this measure should correspond to better adherence on average. For easier interpretation, the release rates were scaled to a 28‐day month. No or very low use of the ring was defined as ≤0.9 mg dapivirine released per month (equivalent to one standard deviation of laboratory measurement error above 0 mg dapivirine released, based on testing of unused rings), whereas target use (i.e. continuous for 28 days) was defined as >4 mg dapivirine per month. Thus, categories of ≤0.9, 0.9 to 4 and >4 mg dapivirine released per month, were used to categorize levels of ring use. Rings that were not returned by the participant were treated as unused. Time that a participant refused a ring was treated as non‐adherent (≤0.9 mg/month).

### 2.3 Statistical analysis

Used ring collection was not initiated until approximately one year after the first participant was enrolled due to logistical considerations. In order to include as much follow‐up as possible, while maintaining comparability between the placebo and active arms, we set each participant’s follow‐up time baseline as the last scheduled visit before ring collection commenced.

Participant characteristics at randomization were summarized according to the highest use category achieved during follow‐up and to whether a participant was still in follow‐up when ring collection and testing commenced (supplementary material). Differences in baseline characteristics were tested using an F test from an ANOVA for means and the Cochran–Mantel–Haenszel test for categorical variables. Analyses were stratified by site.

Descriptive summaries include person‐time according to ring adherence, as defined by the ring use adherence categories as defined above (≤0.9, 0.9 to 4, >4 mg released per month), as well as HIV‐1 incidence within each category (number of infections occurring while in category divided by total person‐years within category). The association between ring use and risk of HIV‐1 infection is summarized as a relative risk reduction (RRR) or 1 minus the hazard ratio estimated using time‐varying Cox models with time to first detection of HIV‐1 RNA as the outcome and women assigned to the placebo arm as the comparison group. Product hold (e.g. for pregnancy or other safety reasons) was rare and was treated as a separate category for both placebo and dapivirine arms and these results were excluded from the presentation. All models were adjusted for the following potential confounders measured at randomization: presence of a curable sexually transmitted infection (*Chlamydia trachomatis*, *Neisseria gonorrhoeae*, *Trichomonas vaginalis*), bacterial vaginosis, alcohol use, marital status, condom use in past week, vaginal intercourse frequency, number of partners, primary partner’s knowledge of ring use, marital status, education, partner’s HIV‐1 status and contraceptive use. These confounders were included as time‐invariant covariates. Endogenous time‐varying confounders were not included in the primary analysis to avoid bias in the estimates of association [18].

Because not all women had rings tested, there is a potential for biased representation of ring use and HIV‐1 incidence in the first year of follow‐up. Therefore, we refit the model censoring HIV testing data prior to month 12. This model thus provided a common time zero for all participants.

Returned rings were collected monthly and at the same time two rapid HIV tests were conducted. Plasma for HIV‐1 RNA testing was collected quarterly and during visits when one or more rapid tests were positive. Therefore, the window around the estimated time of HIV‐1 infection was up to three months. We conducted two exploratory analyses to account for the possibility that HIV‐1 infection measured at a study visit likely reflected HIV‐1 acquisition (and thus ring use) two to twelve weeks prior. Specifically, in the first exploratory analysis, we assumed the adherence level at the time of infection was the lowest adherence measure from the two rings collected prior to HIV‐1 RNA detection. In the second exploratory analysis, we looked at the prior three rings. For both analyses, we did not consider rings used prior to a negative HIV‐1 RNA test. Finally, because there was a statistically significant interaction between age and treatment in the primary intention‐to‐treat results of the trial, all analyses were repeated in the sub‐cohort of women under 25 years of age at baseline. Analyses were conducted using R version 3.3.2 [19].

## 3 RESULTS

A total of 2629 women were enrolled and followed: 15 were infected at randomization or had no further follow‐up, 77 (41 assigned dapivirine, 36 assigned placebo) were lost to follow‐up and 46 (23 assigned dapivirine, 23 assigned placebo) acquired HIV‐1 prior to the first collection of returned rings, leaving 2491 contributing to the present analysis. Therefore, 95% of the women randomized was included in this analysis, retaining 81% of follow‐up time. Participants acquiring HIV‐1 or exiting the study before ring collection had some characteristics suggesting higher HIV‐1 risk (e.g., higher prevalence of STIs at baseline, Table S1). Table 1 shows the baseline characteristics of participants by ring use measurement category. Age, unprotected sex, injectable contraception and oral contraception were all significantly associated with ring use measures.

**Table 1:** Baseline characteristics by categorization of dapivirine release measures throughout follow‐up. Comparisons between the groups are adjusted by site. N (%) reported unless otherwise noted

	Placebo	Rings always 0.9 mg/mo or less	At least one ring > 0.9 mg/mo but no rings > 4mg/mo	At least one ring > 4 mg/mo	*p*‐value
N	1240	50	281	920	
Age (mean (SD))	27.4 (6.3)	25.7 (5.5)	27.6 (6.2)	27.1 (6.1)	0.051
Age group					
18 to 21	226 (18.2)	13 (26.0)	56 (19.9)	186 (20.2)	0.172
22 to 26	412 (33.2)	18 (36.0)	70 (24.9)	290 (31.5)	
27 to 45	602 (48.5)	19 (38.0)	155 (55.2)	444 (48.3)	
*Neisseria gonorrhoeae*	48 (3.9)	1 (2.0)	14 (5.0)	40 (4.3)	0.720
*Trichomonas vaginalis*	86 (6.9)	2 (4.0)	16 (5.7)	66 (7.2)	0.678
*Chlamydia trachomatis*	128 (10.3)	6 (12.0)	37 (13.2)	113 (12.3)	0.316
Partner knows about ring use	802 (64.7)	29 (58.0)	185 (65.8)	58 (63.9)	0.866
Married	534 (43.1)	17 (34.0)	129 (45.9)	369 (40.1)	0.582
Secondary education or higher	1054 (85.0)	42 (84.0)	226 (80.4)	772 (83.9)	0.308
Number of partners (mean (sd))	1.73 (5.73)	2.14 (4.68)	1.83 (6.89)	1.62 (5.28)	0.875
HIV status of primary partner					0.095
Missing	6 (0.5)	0 (0.0)	2 (0.7)	4 (0.4)	
HIV negative	683 (55.1)	31 (62.0)	169 (60.1)	473 (51.4)	
HIV positive	12 (0.1)	1 (2.0)	5 (1.8)	16 (1.7)	
Participant does not know	539 (43.5)	18 (36.0)	105 (37.4)	427 (46.4)	
Bacterial vaginosis	498 (40.2)	20 (40.0)	146 (52.0)	366 (39.8)	0.124
No alcohol use in past seven days					
Unprotected vaginal intercourse reported in past seven days	525 (42.3)	23 (46.0)	127 (45.2)	343 (37.3)	0.033
Condom used at last vaginal sex act	677 (54.6)	28 (56.0)	150 (53.4)	555 (60.3)	0.038
Contraception					
DMPA	535 (43.1)	22 (44.0)	84 (29.9)	373 (40.5)	0.001
IUD (Copper)	159 (12.8)	4 (8.0)	47 (16.7)	106 (11.5)	0.124
Oral pill	121 (9.8)	12 (24.0)	38 (13.5)	92 (10.0)	0.022
Implant	233 (18.8)	6 (12.0)	72 (25.6)	176 (19.1)	0.094
NET‐EN	164 (13.2)	5 (10.0)	27 (9.6)	152 (16.5)	0.041

**Figure 1:**
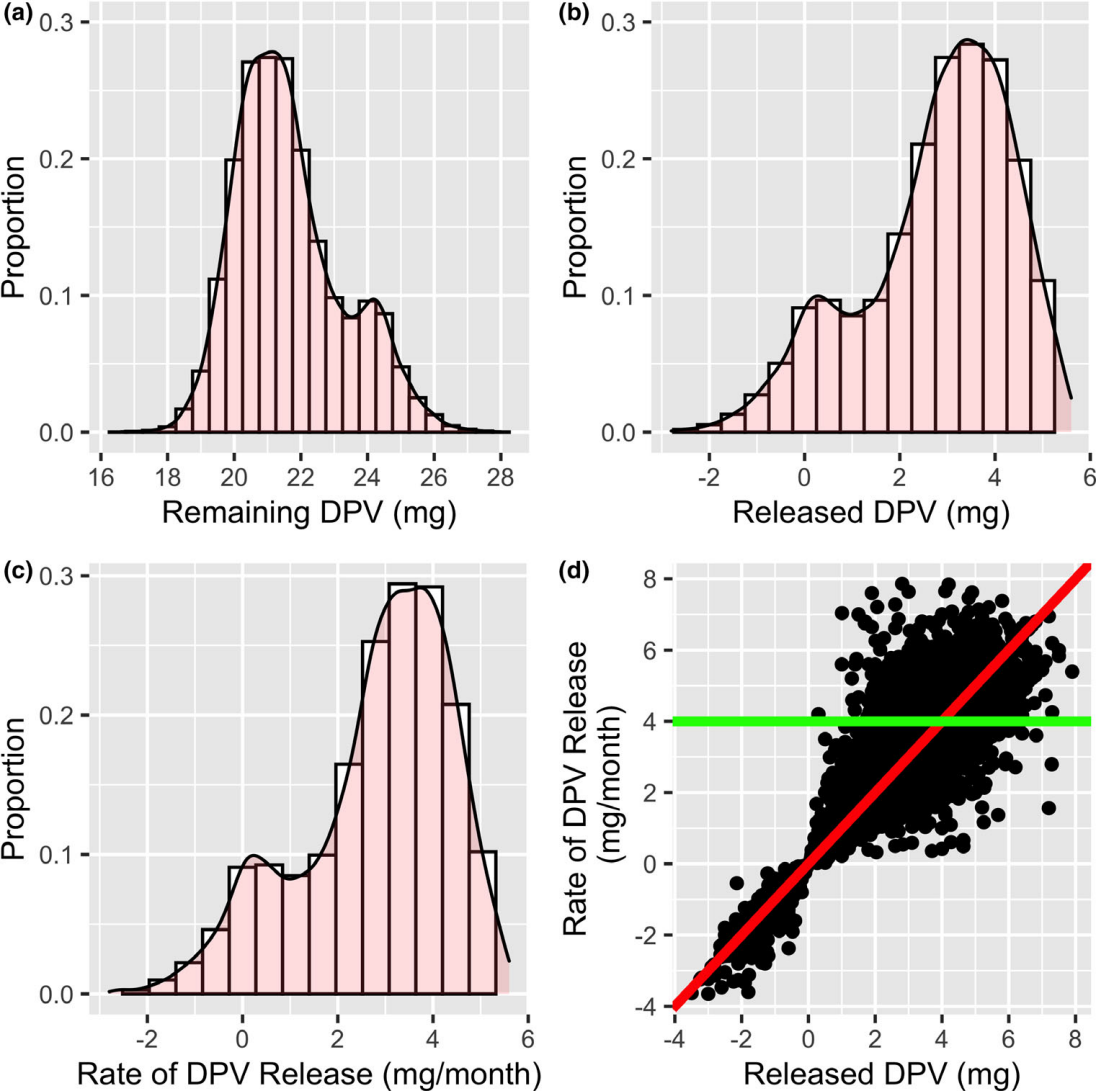
Distributions of remaining dapivirine (DPV) in returned rings **(A)**, DPV released from returned rings **(B)** and rate of DPV release **(C)**. The range of DPV release has a lower limit below 0 due to measurement error. Remaining DPV in returned rings is the measure returned from the laboratory. Released DPV subtracts the lab value from the average load corresponding to the manufacturing batch for that ring. Average rate of release is calculated as the released DPV divided by the time that the participant had the ring. Panel D illustrates the relationship between rate of release and amount released. The red line is the expected relationship (intercept = 0; slope = 1). The green line is the expected rate of release (intercept = 4 mg/28 days; slope = 0) if fully adherent over 28 days. This plot shows how it is important to account for time the ring was available so as to not to potentially mis‐assign participants as fully adherent because the ring was available for longer than the usual 28 days

Figure 1 shows the distribution of dapivirine levels in returned rings in three ways: total amount measured in rings, amount released (manufactured load minus residual amount) and rate of release (the measure of use for the present analysis). Graphical assessment revealed a mixing of multiple distributions: a distribution centred close to what would be expected with no use (mode = 0.34 mg per month released) and a wide range of rates expected to be associated with at least some ring use.

Using all returned ring data, HIV‐1 incidence was highest in women during periods for which residual dapivirine levels indicated non‐use (i.e. residual levels ≤0.9 mg released per month) and that incidence was similar to incidence among women assigned placebo (Table 2). In contrast, incidence was lower during periods with evidence of use. Specifically, residual dapivirine ring levels suggestive of at least some use (i.e. >0.9 mg released over a month) were associated with a 48% (95% CI: 21, 66) reduction in HIV‐1 acquisition risk (*p* = 0.002). In exploratory analyses and analyses that reset the time zero to the Month 12 visit, similar results were observed, with a clearer dose‐response increase in HIV‐1 protection with more dapivirine release – that is greater protection with >4 mg released compared to 0.9 to 4 mg released. Using all available data, the exploratory analyses estimated 75% HIV‐1 relative risk reduction with >4 mg released, 85% (95% CI: 51, 95) when considering the lowest dapivirine residual data from the prior three months and 91% when limited to data after Month 12.

**Table 2:** Incidence and risk reduction by arm and amount of drug released from the returned rings

	Placebo	Non‐adherent	Any adherence (>0.9 mg/mo)	0.9 to 4 mg/mo	Target release rate (>4mg/mo)
**All available data**					
Incidence (95% CI)	4.2 (3.3, 5.3)	4.8 (2.7, 8.0)	2.3 (1.6, 3.2)	2.1 (1.4, 3.2)	2.6 (1.4, 4.5)
# events/p‐y	71/1678	13/269	32/1413	21/989	11/423
RRR		3.4%	48.0%	50.3%	43.0%
(95% CI)		(−76.5, 47.1)	(20.8, 65.9)	(18.7, 69.6)	(−8.7, 70.1)
*p*‐value		0.911	0.002	0.005	0.088
*Exploratory analyses*					
RRR associated with lowest adherence level observed within prior two months or since last negative if less than two months prior		3.6%	48.2%	35.9%	74.6%
(95% CI)		(−73.1, 46.3)	(21.2, 66.0)	(−0.4, 59.0)	(36.8, 89.8)
*p*‐value		0.902	0.002	0.052	0.003
RRR associated with lowest adherence level observed within prior two months or since last negative if less than three months prior		7.3%	47.9%	30.5%	84.8%
(95% CI)		(−66.5, 48.4)	(20.6, 65.8)	(−7.6, 55.1)	(51.3, 95.2)
*p*‐value		0.799	0.002	0.102	0.001
**Month 12 baseline**					
Incidence (95% CI)	4.3 (3.1, 5.7)	4.2 (1.9, 8.2)	1.8 (1.0, 2.9)	2.0 (1.0, 3.4)	1.3 (0.4, 3.4)
# events/p‐y	41/959	7/167	14/798	11/563	3/235
RRR		7.7%	59.4%	54.3%	71.3%
(95% CI)		(−108.8, 59.2)	(25.0, 78.1)	(10.2, 76.7)	(6.4, 91.2)
*p*‐value		0.85	0.004	0.023	0.039
*Exploratory analyses*					
RRR associated with lowest adherence level observed within prior two months or since last negative if less than two months prior		6.3%	60.0%	46.5%	90.6%
(95% CI)		(−103.5, 56.8)	(26.1, 78.3)	(−0.7, 71.6)	(31.2, 98.7)
*p*‐value		0.870	0.003	0.053	0.020
RRR associated with lowest adherence level observed within prior three months or since last negative if less than three months prior		7.1%	59.6%	46.1%	90.5%
(95% CI)		(−101.5, 57.1)	(25.5, 78.1)	(−1.5, 71.4)	(30.7, 98.7)
*p*‐value		0.853	0.004	0.056	0.020
**All available data, under 25 at randomization**					
Incidence (95% CI)	5.7 (4.1, 7.9)	7.9 (4.1, 14.0)	4.1 (2.6, 6.2)	3.8 (2.1, 6.3)	4.7 (2.2, 8.9)
# events/p‐y	35/612	10/126	21/510	13/341	8/169
Effectiveness		−34.3%	33.2	37.2%	25.1%
(95% CI)		(−176.2, 34.7)	(−16.6, 61.7)	(−20.6, 67.3)	(−66.0, 66.2)
*p*‐value		0.423	0.156	0.162	0.476
*Exploratory analyses*					
RRR associated with lowest adherence level observed within prior two months or since last negative if less than two months prior		−20.9%	34.1%	18.3%	64.3%
(95% CI)		(−159.6, 41.4)	(−14.6, 62.1)	(−48.2, 54.9)	(−2.8, 87.6)
*p*‐value		0.608	0.140	0.507	0.056
RRR associated with lowest adherence level observed within prior three months or since last negative if less than three months prior		−18.9%	33.9%	13.4%	73.1%
(95% CI)		(−145.2, 42.4)	(−15.0, 62.1)	(−55.4, 51.7)	(10.7, 91.9)
*p*‐value		0.639	0.143	0.629	0.032
**Month 12 baseline, under 25 at randomization**					
Incidence (95% CI)	5.9 (3.8, 8.9)	7.6 (3.1, 15.6)	2.4 (1.1, 4.7)	2.5 (1.0, 5.5)	2.2 (0.4, 6.9)
# events/p‐y	21/354	6/79	7/291	5/199	2/93
RRR		−44.5%	53.7%	50.5%	60.2%
(95% CI)		(−271.8, 43.9)	(−12.8, 81.0)	(−35.9, 82.0)	(−77.5, 91.1)
*p*‐value		0.45	0.09	0.44	0.17
*Exploratory analyses*					
RRR associated with lowest adherence level observed within prior two months or since last negative if less than two months prior		−19.7%	55.4%	42.8%	80.7%
(95% CI)		(−207.2, 53.3)	(−8.1, 81.2)	(−46.5, 77.7)	(−48.9, 97.5)
*p*‐value		0.708	0.074	0.245	0.111
RRR associated with lowest adherence level observed within prior three months or since last negative if less than three months prior		−18.6%	55.2%	42.4%	80.9%
(95% CI)		(−204.3, 53.8)	(−8.6, 81.1)	(−47.3, 77.5)	(−46.4, 97.5)
*p*‐value		0.723	0.076	0.249	0.111

For each subset of data, relative risk reduction (RRR = 1 – hazard ratio) was calculated according to the primary analysis with the HIV endpoint occurring at the time of the first detection of RNA and using two exploratory analyses labelled as two months (where infection was assumed to occur at the lowest adherence observed in the previous two months, if a negative RNA test had not been conducted in the month in between) and three months (where infection was assumed to occur at the lowest adherence observed since the last negative test; if there was not a negative test in the preceding three months, infection was assumed to occur at the lowest observed adherence level in the previous three months).

A similar pattern in risk reduction was seen in the subgroup of women under 25 years old at randomization – specifically, lower HIV‐1 incidence and higher protection associated with greater objective evidence suggesting adherence, although only one result was statistically significant in this smaller group. Notably, while approximately half of the infections in the placebo arm occurred in women under 25 years of age (35 of 71), 10 of 13 infections in the non‐adherent group occurred in the younger women (incidence rate 7.9 per 100 person‐years; 95% CI: 4.1, 14.0). Among women under 25 years of age, point estimates for HIV‐1 protection (target use vs. placebo) were 25% (95% CI: −66, 66; *p* = 0.48) overall and 73% (95% CI: 11, 92; *p* = 0.03) for the lowest residual dapivirine quantified from the prior three months. In the subset with month 12 as baseline, the corresponding point estimates were 60% (95% CI: −78, 91; *p* = 0.17) and 81% (95% CI: −46, 98; *p* = 0.11).

## 4 DISCUSSION

In this secondary analysis from a randomized, placebo‐controlled trial of the dapivirine vaginal ring, objective measures suggesting adherence to the ring were significantly correlated with HIV‐1 protection. There was some evidence to suggest a dose‐response effect, and HIV‐1 protection was robust to adjustment for potential confounding factors. Exploratory analyses designed to estimate the highest potential efficacy showed substantial increases in protection. Results were generally consistent when limited to the subgroup of women <25 years of age, who tended to have lower adherence. Taken together, these analyses indicate the potential for a substantial reduction in the risk of HIV‐1 acquisition – greater than 50%, and potentially as high as 75% to 91% – in women who use, and ideally consistently wear, the dapivirine vaginal ring.

Our findings have similarities to those of prior studies of oral tenofovir‐based PrEP, which found substantially lower HIV‐1 incidence rates when objective measures of adherence indicated higher use of the prevention product. For example the iPrEx trial showed a 42% reduction in HIV‐1 risk in intention‐to‐treat analyses compared to placebo, but >90% HIV‐1 protection in analyses accounting for product use as measured by tenofovir concentrations in blood samples [20]. Several features of the dapivirine vaginal ring make assessment of adherence potentially more challenging than for oral PrEP. First, in oral PrEP studies, there was little incentive for “white coat dosing” (i.e. use just before a clinic visit) and low levels of “white coat dosing” reported in women [21]; however, in ASPIRE, women were expected to return to the clinic with the dapivirine vaginal ring in place, resulting in an expectation of at least some use around the time of a clinic visit. Second, there are no directly observed therapy studies of the dapivirine vaginal ring to correlate drug levels in returned rings to actual product use, while such studies were important for understanding objective measures of PrEP use [10, 22]; even the >4 mg released per month estimate was derived from phase I/II studies in which the degree of ring use was self‐reported. Third, the amount of dapivirine expected to be released even with consistent use is approximately 16% to 18% of the total drug loaded into each ring, which could result in misclassification when levels take into account allowable error in the laboratory measurement of dapivirine levels. Nevertheless, our results consistently suggested protection across multiple analyses.

There are several limitations to this analysis. First, returned rings were not tested at all visits for the entire cohort, leading to this sub‐cohort, a convenience sample from the overall trial population. Adherence was likely lower in the period before ring collection [23] and therefore, the missing early data may result in bias in the final analysis. For example if participants with a higher propensity to be non‐adherent are also at higher risk, they may have acquired HIV‐1 before ring collection, diluting the pool of highest‐risk, lowest‐adherent participants, resulting in an underestimate of incidence during periods of non‐ or low adherence, an effect which would make our results underestimates of HIV‐1 protection. Although we adjusted for predictors of adherence and risk, most importantly age and site, unknown potential confounding potentially remains. Due to variability inherent in manufacturing and testing, a fraction of rings assigned to each category might have been more accurately assigned to another category. However, typically, this type of misclassification biases results to the null. This may be especially true for the highest ring use category as that group is likely to have low use rings misclassified as high use and to have lost high use rings to the lower use group.

Although we presented cut points for analysis of residual dapivirine levels (≤0.9, 0.9 to 4, >4 mg released on average over a month), we have not identified a threshold for HIV‐1 protection. Similarly, for oral PrEP, while one threshold of protection has been suggested for MSM [10, 20], there is no universal threshold yet identified. Additional challenges specific to the dapivirine vaginal ring and to this analysis make defining a threshold of protection difficult. We found that the relationship between dapivirine release and risk was stronger when baseline was set to the Month 12 visit, an effect that may be real or due to informatively missing data in the first year, as described above. Additionally, theoretically, women do not need to be fully adherent to be fully protected. For example if a woman only has vaginal intercourse once in a month and the ring is in place in a wide enough window around that one point in time, she could be protected, but remaining drug levels may indicate inconsistent use. Nevertheless, the near zero estimate of the low dapivirine release rate group compared to placebo provides evidence of a potential causal relationship in the estimate of effectiveness when comparing rates greater than 0.9mg over a month to HIV‐1 rates in those assigned to placebo [24].

## 5 CONCLUSIONS

Alongside oral PrEP, the dapivirine vaginal ring provides another option for women to protect themselves from HIV‐1. Moreover, like for oral PrEP, the degree of HIV‐1 protection with greater adherence exceeds 50% and may be as high as 75% to 91%. As seen in family planning where contraceptive choices have diversified over time and population coverage has increased [25], providing new options for women to protect themselves engages women who were not successfully accessing or using the previously available options. Rings with a higher dapivirine load and added contraception are under development and could provide longer or possibly better HIV‐1 prevention (ClinicalTrials.gov #NCT03234400). The dapivirine vaginal ring has the potential to similarly fill an important place in offering women choices for successfully achieving HIV‐1 prevention.

## COMPETING INTEREST

The authors declare no competing interest.

## AUTHORS’ CONTRIBUTIONS

ERB, SLH and JMB designed the study. ERB analysed the data. ERB and JMB drafted the paper, and all authors have read, edited and approved the final manuscript.

## Supporting information


**Table S1.** Baseline characteristics by inclusion in analysis cohort (exited follow‐up before vs. after ring collection commenced). Comparisons between the groups are adjusted by site. N (%) reported unless otherwise noted
