# Supplementary material for: Greater dapivirine release from the dapivirine vaginal ring is correlated with lower risk of HIV‐1 acquisition: a secondary analysis from a randomized, placebo‐controlled trial
Source: J Int AIDS Soc. 2020 Nov 18;23(11):e25634. doi: 10.1002/jia2.25634 (PMC7673220; doi:10.1002/jia2.25634)
Supplement: Supplementary file 1 — Table S1. Baseline characteristics by inclusion in analysis cohort (exited follow‐up before vs. after ring collection commenced). Comparisons between the groups are adjusted by site. N (%) reported unless otherwise noted [file JIA2-23-e25634-s001.docx]

Table S1 Baseline characteristics by inclusion in analysis cohort (exited follow-up before versus after ring collection commenced). Comparisons between the groups are adjusted by site. N (%) reported unless otherwise noted.

|  | Excluded | Included | p-value |
| --- | --- | --- | --- |
| N | 123 | 2491 |  |
| Dapivirine arm (%) | 64 (52.0) | 1244 (49.9) | 0.74 |
| Age (mean (sd)) | 25.46 (5.53) | 27.32 (6.21) | 0.07 |
| Age group |  |  | 0.71 |
| 18-21 | 31 (25.2) | 481 (19.3) |  |
| 22-26 | 50 (40.7) | 790 (31.7) |  |
| 27-45 | 42 (34.1) | 1220 (49.0) |  |
| *Neisseria gonorrhoeae* | 6 ( 4.9) | 103 ( 4.1) | 0.71 |
| *Trichomonas vaginalis* | 10 ( 8.1) | 170 ( 6.8) | 0.65 |
| *Chlamydia trachomatis* | 28 (22.8) | 284 (11.4) | 0.01 |
| Partner knows about ring use | 67 (54.5) | 1604 (64.4) | 0.12 |
| Married | 20 (16.3) | 1049 (42.1) | 0.16 |
| Secondary education or higher | 117 (95.1) | 2094 (84.1) | 0.41 |
| Number of partners (mean (sd)) | 1.17 (0.62) | 1.71 (5.69) | 0.72 |
| HIV status of primary partner |  |  | 0.65 |
| Missing | 1 ( 0.8) | 12 ( 0.5) |  |
| HIV negative | 79 (64.2) | 1356 (54.4) |  |
| HIV positive | 1 ( 0.8) | 34 ( 1.4) |  |
| Participant does not know | 42 (34.1) | 1089 (43.7) |  |
| Bacterial vaginosis | 56 (45.5) | 1030 (41.3) | 0.45 |
| No alcohol use in past 7 days | 105 (85.4) | 2191 (88.0) | 0.14 |
| Unprotected vaginal intercourse reported in past 7 days | 28 (22.8) | 1018 (40.9) | 0.05 |
| Condom used at last vaginal sex act | 83 (67.5) | 1410 (56.6) | 0.43 |
| Contraception |  |  |  |
| DMPA | 54 (43.9) | 1014 (40.7) | 0.86 |
| IUD (Copper) | 7 ( 5.7) | 316 (12.7) | 0.71 |
| Oral pill | 22 (17.9) | 263 (10.6) | 0.66 |
| Implant | 10 ( 8.1) | 487 (19.6) | 0.97 |
| NET-EN | 28 (22.8) | 348 (14.0) | 0.71 |
